# Challenges in microbiological identification of aerobic bacteria isolated from the skin of reptiles

**DOI:** 10.1371/journal.pone.0240085

**Published:** 2020-10-19

**Authors:** Maria Brockmann, Heike Aupperle-Lellbach, Michaela Gentil, Anton Heusinger, Elisabeth Müller, Rachel E. Marschang, Michael Pees

**Affiliations:** 1 LABOKLIN GmbH & Co. KG, Bad Kissingen, Germany; 2 Clinic for Birds and Reptiles, University of Leipzig, Leipzig, Germany; University of San Agustin, PHILIPPINES

## Abstract

**Introduction:**

Bacterial pathogens are often involved in dermatitis in reptiles. Exact identification of reptile-specific but otherwise uncommon bacterial species may be challenging. However, identification is crucial to evaluate the importance of the detected bacterial species.

**Objective:**

The aim of this study was to assess the number of aerobic bacterial isolates cultured from skin-derived samples of reptiles which were not reliably identified by Matrix-Assisted Laser Desorption/Ionization Time-of-Flight Mass Spectrometry (MALDI-TOF MS), and to determine their identity.

**Material and methods:**

Routine bacterial diagnostics were performed on 235 skin samples, and 417 bacterial isolates were analysed by MALDI-TOF MS. The isolates were grouped into categories based on their first score: category I (≥ 2.00), category II (≥ 1.70 and < 2.00), and category III (< 1.70). Isolates from category III were further investigated by 16S rRNA gene sequencing and the following criteria were applied: query cover 100%, e-value rounded to 0.0 and sequence identity (%) > 98.00% for genus identification, and > 99.00% for species identification.

**Results:**

The majority of bacterial isolates were in category I (85.1%) or category II (8.4%). In category III (6.5%) results achieved at first by MALDI-TOF MS corresponded to the results of the molecular analysis in 8.0% of isolates at the species level and in 24.0% at the genus level. Bacterial isolates classified as category III were heterogenic in genus (e.g. *Chryseobacterium*, *Devriesea*, *Pseudomonas*, *Staphylococcus*, *Uruburuella*), and some have only been described in reptiles so far.

**Conclusions:**

Most of the aerobic bacterial isolates cultured from reptile skin achieved high scores by MALDI-TOF MS. However, in the majority of category III isolates MALDI-TOF MS results were different from those of the molecular analysis. This strengthens the need to carefully examine low-scored results for plausibility and to be familiar with the occurrence and morphology of relevant reptile-specific bacterial species (e.g. *Devriesea agamarum*) as well as with the limits of the database used.

## Introduction

Dermatitis is a common presentation in reptiles in veterinary practice. Animals affected may be presented with signs of abscesses, blisters, crusts, edema, ecchymoses/petechiae, erythema, loose scales or scutes and/or ulcerated, necrotic skin [1,2]. Dermatitis is often associated with underlying husbandry issues [1,2]. It is painful and can often be seen in conjunction with systemic disease, particularly septicaemia [3,4]. Since various bacterial genera, often gram-negative genera like *Aeromonas*, *Citrobacter*, or *Serratia*, are commonly involved [5], exact identification is essential to evaluate the importance of the detected bacterial species and develop a treatment plan. Numerous techniques are available for the identification of bacterial colonies. Historically, this has been based on morphology, staining properties, as well as biochemical qualities [6]. With the advent of molecular techniques, sequencing of ribosomal ribonucleic acid (rRNA) genes became a popular tool for bacterial identification and determination of phylogenetic relationships [7–9]. The 16S rRNA gene is universal and highly conserved, but contains enough variable regions to discriminate between different bacterial genera and even species in many cases [10,11]. This makes it a universal and useful target for identifying bacterial species. With 16S rRNA gene sequencing, it is possible to analyse even slow-growing or uncultivable bacteria [6,10]. In recent decades, 16S rRNA gene sequencing has still been used widely for bacterial identification, including those from reptiles [12,13]. The use of this technique in daily bacteriological routine diagnostics is nevertheless limited due to its costs [14,15], the time needed in comparison to e.g. Matrix-Assisted Laser Desorption/Ionisation Time-of-Flight Mass Spectrometry (MALDI-TOF MS) and the fact that it is not always possible to discriminate species within some genera using 16S rRNA gene sequencing [8,16].

MALDI-TOF MS is currently used routinely for bacteriological examination in both, human and veterinary medicine, and has proven to be a fast and cost effective method [17,18]. With this technique, protein mass spectra of a bacterial colony can be examined and compared to a database of profiles for defined species. This reference database is usually supplied by the manufacturer. The most probable results appear with a score based on a comparison of the test spectrum and the reference spectrum [19]. These scores are used to judge the reliability of the result. Usually, scores ≥ 2.00 are considered credible at the genus level and probable at the species level. Scores ≥ 1.70 and < 2.00 are considered reliable at the genus level but insufficiently dependable at the species level, and scores < 1.70 are not considered credible [20,21]. Reliability of this method is often evaluated by comparison with 16S rRNA gene sequencing [21,22].

Despite the advantages of MALDI-TOF MS for routine identification of bacterial isolates, several papers have revealed problems with the identification of various isolates using MALDI-TOF MS [18,23]. Identification has been found to be unreliable at the species level for several genera [20,24]. This may be due to a high resemblance between individual species within a genus, as has been documented for example for some *Citrobacter* species [25]. These problems are usually known to the manufacturer, and a comment appears if one of these species is considered as a probable result by MALDI-TOF MS, even if an appropriate score is achieved. Nevertheless, results considered unreliable (score < 1.70) occur frequently in veterinary diagnostics [18] and bacteria isolated from minor species such as reptiles seem especially frequently affected [23]. In the worst case this could lead to treatment errors affecting the health of the patient.

To our knowledge, there are currently no studies providing results on the frequency of unreliable identification of aerobic bacterial isolates derived exclusively from dermatological samples of reptiles by MALDI-TOF MS. The aim of this study was to assess the number of aerobic bacterial isolates cultured from skin-derived samples of reptiles which were not reliably identified by MALDI-TOF MS. It was then the aim to determine the genus and possible species of unreliably identified isolates using 16S rRNA gene sequencing. Based on these findings, a further goal was to evaluate whether some bacterial species cause identification problems more frequently and if scores below 1.70 should necessarily be followed up by a reevaluation via 16S rRNA gene sequencing. A guideline on how to proceed with MALDI-TOF MS data for reptile skin-derived aerobic bacterial isolates in order to obtain reliable results is suggested.

## Material and methods

### Collection of samples and bacteriological examination

Between January 2019 and December 2019 skin samples from 235 reptiles (136 chelonians and 99 squamates, including 38 snakes) were examined by aerobic bacterial culture in routine diagnostics. This was a retrospective study based on samples submitted for veterinary diagnostic testing, therefore no animal research ethics approval was necessary. Information on host species, if provided by the submitting veterinarian, is made available in S1 Table “Supplemental information on host species in alphabetical order”. Samples were derived from skin, cutaneous abscesses, and carapace or plastron. Clinical signs were not a criterion for inclusion in this study, however, in those cases in which information was available, most animals had clinical signs of skin disease.

Samples were inoculated onto Columbia agar with defibrinated sheep blood (Becton Dickinson GmbH, Heidelberg, Germany/Oxoid GmbH, Wesel, Germany) and incubated at 36°C for 18–24 hours. Incubation in aerobic atmosphere was extended for another 18–24 hours if no growth was detected. To gain pure cultures, single colonies were picked out of a mixed culture based on different growth morphology and inoculated onto a separate agar plate. Identification was based on growth on selective agar plates, biochemical parameters and MALDI-TOF MS. MALDI-TOF MS (Microflex LT/SH, Bruker Daltonics, Bremen, Germany) was used to examine 417 bacterial isolates in direct transfer. For this, material from single colonies of the culture in question was randomly picked with a toothpick, a thin layer was applied to a target, overlaid with 1.0μl matrix (HCCA; α-Cyano-4-hydroxycinnamic acid), and dried at room temperature before examination by MALDI-TOF MS. The following databases were used for isolate identification: MALDI Biotyper Reference Library: MBT Compass Library Revision E, MBT 7854 MSP Library and, beginning in December 2019, Revision F, MBT 8468 MSP Library (Bruker Daltonics, Bremen, Germany).

Agreement between the created spectrum and reference spectra provided by the Bruker database is expressed by a confidence score. The first score accorded each isolate was used to divide the isolates into three categories: category I (score ≥ 2.00), category II (score ≥ 1.70 and < 2.00) and category III (score < 1.70). Category I isolates, along with category II isolates, were not examined further, as these were considered reliably identified to at least the genus level based on MALDI-TOF MS [24,26] and morphologic and biochemical characterization. Category III isolates were included in the further study. The top three MALDI-TOF MS results (bacterial species) were recorded and compared with the results of the molecular analysis. Several bacterial species have more than one corresponding entry in the database. The first three bacterial species sorted by highest score were checked for doublings which were marked. If there were any, the subsequent bacterial species were taken into account. Thus, always the top three unique bacterial species listed by the database were considered.

### Preservation of pure cultures

Microbank^™^ (Pro Lab Diagnostics, Richmond Hill, Canada) was used for preservation of pure cultures of category III isolates. Isolates were frozen at -18°C and kept at that temperature for up to twelve months. After defrosting, individual isolates were inoculated onto Columbia sheep blood agar (Becton Dickinson GmbH, Heidelberg, Germany/Oxoid GmbH, Wesel, Germany) to be rechecked for growth and pure culture.

### Molecular analysis

Pure cultures were swabbed and swabs were incubated in 750μl lysis buffer (MagNA Pure DNA Tissue Lysis Buffer, Roche, Mannheim, Germany) and 75μl proteinase K (proteinase K, lyophilisiert, ≥ 30 U/mg, Carl Roth GmbH und Co KG, Karlsruhe, Germany) for one hour at 65°C. From this, 200μl were utilized for automated nucleic acid extraction using the MagNAPure 96 DNA and Viral NA Small Volume Kit (Roche, Mannheim, Germany) according to the manufacturer’s instructions. The resulting DNA was eluted in a volume of 100μl. The isolated DNA was kept at a temperature of -18°C until the PCR was performed. Two universal and commonly utilized [27,28] primers, 27F: 5´AGA GTT TGA TCM TGG CTC AG 3´ and 926R: 5´CCG TCA ATT CCT TTR AGT TT 3´ (Eurofins MWG Operon, Ebersberg, Germany), were used to amplify a portion of the 16S rRNA gene (expected amplicon size was approximately 899 base pairs (bp)). Reactions included 1.0μl of each primer (10μM), 0.5μl DFS Hot Taq DNA Polymerase (5U/μl), 0.5μl dNTP-Mix (4x10mM), 2.5μl 10X reaction buffer (Polymerase, dNTP-Mix and reaction buffer from DFS Hot Taq DNA Polymerase-Kit, GeneON, Ludwigshafen, Germany), and 5.0μl template DNA in a total volume of 25μl. A Biometra TOne thermocycler (Analytik Jena, Jena, Germany) was used for the amplification, which consisted of an initial denaturation step at 95°C for 10 minutes, followed by 35 rounds of denaturation at 95°C for 60 seconds, annealing at 52°C for 60 seconds, and extension at 72°C for 90 seconds, with a final extension at 72°C for 10 minutes. PCR grade water (Roche, Mannheim, Germany) was used as a negative control and DNA prepared from a *Staphylococcus aureus* isolate as a positive control for each reaction.

Amplification was verified using gel electrophoresis (Invitrogen by Thermo Fisher Scientific, Darmstadt, Germany).

#### 16S rDNA sequencing and analysis

PCR-products were purified using the DyeEx 2.0 Spin Kit and the MinElute PCR Purification Kit (Qiagen, Hilden, Germany), and Sanger sequencing (ABI PRISM 3130 XL Genetic Analyser, Applied Biosystems) was performed in both directions using the primers 27F and 926R described above. Sequence contigs were manually controlled and formed from the forward and reverse ends using BioEdit 7.2.5 software [29].

Alignment of the sequences was executed by Basic Local Alignment Search Tool (BLAST) (https://blast.ncbi.nlm.nih.gov/Blast.cgi) using the standard database. To provide reliability, the query cover had to be 100%, sequence identity (%) > 98.00% for genus identification, and > 99.00% for species identification, and the e-value rounded to 0.0 by BLAST itself, as this indicates highest significance of the match [30]. If these criteria were fulfilled by multiple results indicating different bacterial species, the top result was chosen. If two or more results indicating different bacterial species had the same sequence identity (%), all of those were listed. If the sequence identity (%) was > 98.00% and ≤ 99.00% only the genus was considered reliable. However, the top bacterial species result was noted in brackets.

## Results

No bacterial growth was observed in 15 (6.3%) of the 235 samples tested. In total, 443 bacterial isolates were obtained from the 220 positive samples with one to five isolates obtained from individual samples.

### Analysis of the isolates by MALDI-TOF MS

Of the 443 bacterial isolates obtained, 26 isolates (19 isolates of aerobic spore-forming bacteria, one *Enterococcus* sp., three *Escherichia* spp., one species of alpha haemolytic streptococci, one *Proteus* sp., one *Staphylococcus* sp.) were identified without the use of MALDI-TOF MS and therefore excluded from this study. Of the 417 isolates, which were examined via MALDI-TOF MS, the majority (355 isolates, 85.1%) were in category I, or category II (35 isolates; 8.4%). The remaining 27 isolates (6.5%) were classified in category III and thus were included in the molecular analysis.

### Molecular analysis

Molecular analysis results were obtained from 25 of 27 category III isolates (Table 1). These isolates were revealed to be genetically heterogeneous. Of the 25 isolates, 15 were gram-positive, belonging to ten different genera. The remaining ten isolates were gram-negative, belonging to nine different bacterial genera. For some isolates, BLAST analysis resulted in several different bacterial species, which achieved similar probabilities for identity in terms of percentage noted (Table 1). This occurred for instance for different species within the genus *Pseudomonas*, in this case with a score of 100% identity achieved. Molecular analysis was not possible for one isolate identified as *Acinetobacter* sp. (score 1.65), and one isolate later identified as *Chryseobacterium* sp. (first examination: no result) by MALDI-TOF MS, due to contamination for the one and a lack of regrowth after deep-freezing for the other.

**Table 1:** Results of MALDI-TOF MS and 16S rRNA gene sequencing of bacterial isolates assigned to category III.

Sample No.	Host species	MALDI-TOF MS: First three bacterial species results provided (score)	16S rRNA gene result: Bacterial species provided with highest sequence identity	GenBank accession number of reference sequence	Sequence identity (%)	Querylength (bp)	Level of agreement
1	*Trachemys scripta*	No result	*Achromobacter xylosoxidans*	CP002287.1	100.00	765	None
2	*Testudo marginata*	*Acinetobacter calcoaceticus* (1.65) *Acinetobacter johnsonii* (1.55) *Acinetobacter bouvetii* (1.54)	*Acinetobacter haemolyticus*	KJ806420.1	99.86	712	Genus
3	Uromastycinae	*Bacillus humi* (1.60) *Staphylococcus vitulinus* (1.41) *Pseudarthrobacter oxydans* (1.33)	*Bacillus endophyticus* *Bacillus filamentosus*	MT487659.1 MT479162.1	99.56 99.56	685	Genus
4	*Graptemys pseudogeographica*	No result	*Bacillus alkalisediminis* *Bacillus amyloliquefaciens* *Bacillus aryabhattai* *Bacillus cabrialesii* *Bacillus cereus* *Bacillus subtilis* *Bacillus tequilensis* *Bacillus velezensis*	MT534562.1 MT588736.1 MT588735.1 MT377907.1 MT394923.1 MT590663.1 MT588717.1 MT377909.1	100.00 100.00 100.00 100.00 100.00 100.00 100.00 100.00	725	None
5	*Furcifer pardalis*	*Kokuria rosea* (1.40)^b^ *Arthrobacter parietis* (1.28) *Kytococcus sedentarius* (1.25)	*Brachybacterium conglomeratum* *Brachybacterium paraconglomeratum*	MT386331.1 MH929581.1	99.42 99.42	686	None
6	Chelonian (unspecified)	*Chryseobacterium indologenes* (1.60) *Chryseobacterium wanjuense* (1.60) *Chryseobacterium joostei* (1.56)	*Chryseobacterium culicis*	JF899295.1	99.87	787	Genus
7	Chelonian (unspecified)	*Lactobacillus diolivorans* (1.21) *Corynebacterium bovis* (1.19) *Pseudomonas veronii* (1.14)	*Cloacibacterium normanense*	MK294295.1	99.86	727	None
8	*Testudo hermanni*	*Pseudomonas luteola* (1.36) *Arthrobacter monumenti* (1.34) *Citrobacter freundii* (1.34)	*Corynebacterium freneyi*^a^ *Corynebacterium xerosis*	KM378610.1CP046322.1	100.00 100.00	762	None
9	*Graptemys pseudogeographica*	*Exiguobacterium aurantiacum* (1.61) *Bacillus alcalophilus* (1.34) *Staphylococcus aureus* (1.33)	*Deinococcus aquaticus*	MH504182.1	100.00	799	None
10	Chelonian (unspecified)	*Lactobacillus graminis* (1.27) *Elizabethkingia meningoseptica* (1.20) *Stenotrophomonas nitrireducens* (1.06)	*Deinococcus indicus*	NR_118357.1	99.48	763	None
11	*Testudo hermanni*	*Lactobacillus graminis* (1.27) *Tissierella praeacuta* (1.18) *Listeria ivanovii* (1.16)	*Desemzia* sp. [*Desemzia incerta*]	NR_119259.1	98.41	752	None
12	*Brachylophus fasciatus*	*Lactobacillus brevis* (1.07) *Lactobacillus delbrueckii* (1.05) *Xanthomonas hyacinthi* (1.02)	*Devriesea agamarum*	LN849456.1	99.88	800	None
13	Uromastycinae	*Staphylococcus hominis* (1.23) *Flavobacterium pectinovorum* (1.16) *Streptomyces violaceoruber* (1.15)	*Devriesea agamarum*	LN849456.1	100.00	800	None
14	Uromastycinae	*Vibrio vulnificus* (1.17) *Bacillus infantis* (1.15) *Cutibacterium acnes* (1.15)	*Devriesea agamarum*	LN849456.1	99.87	745	None
15	Chelonian (unspecified)	*Lactobacillus* *kalixensis* (1.25) ^b^ *Arthrobacter roseus* (1.25) *Exiguobacterium* sp. (1.23)	*Exiguobacterium acetylicum*	MN704794.1	99.63	800	Genus listed in top MALDI-TOF MS results
16	*Trachemys scripta elegans*	*Staphylococcus pasteuri* (1.30) *Listeria grayi* (1.25) ^b^ *Staphylococcus warneri* (1.21)	*Exiguobacterium* sp. [*Exiguobacterium acetylicum*]	MN314587.1	98.63	800	None
17	*Sternotherus odoratus*	*Pseudomonas segetis* (1.34) *Arthrobacter gandavensis* (1.32) *Arthrobacter ramosus* (1.32)	*Micrococcus luteus* *Micrococcus yunnanensis*	CP033200.1 MN421481.1	99.88 99.88	819	Family listed in top MALDI-TOF MS results
18	*Egernia stokesii*	*Ochrobactrum gallinifaecis* (1.63) *Ochrobactrum grignonense* (1.57) *Lactobacillus sharpeae* (1.24)	*Ochrobactrum lupini* *Ochrobactrum pseudogrignonense* *Paenibacillus* sp^a^ *Pantoea* sp^a^	JF509158.1 MN889385.1 MH558371.1 MH558373.1	100.00 100.00 100.00 100.00	761	Genus
19	*Testudo hermanni*	*Pseudomonas corrugata* (1.53) *Pseudomonas savastanoi* (1.50) *Pseudomonas extremorientalis* (1.45)	*Pseudomonas oryzihabitans* *Pseudomonas putida* *Pseudomonas reidholzensis*	MT033070.1 MT192452.1 MT370524.1	100.00 100.00 100.00	794	Genus
20	*Corytophanes cristatus*	No result	*Serratia marcescens*	MF171123.1	99.87	789	None
21	Uromastycinae	*Sporosarcina luteola* (1.42) *Sphingomonas faeni* (1.38) *Arthrobacter koreensis* (1.38)	*Sporosarcina thermotolerans*	KT719638.1	100.00	802	Genus
22	*Furcifer pardalis*	*Staphylococcus kloosii* (1.36) *Micrococcus luteus* (1.32) *Staphylococcus cohnii* (1.30)	*Staphylococcus kloosii*	MN733166.1	99.61	766	Species
23	*Egernia stokesii*	*Stenotrophomonas rhizophila* (1.67) *Stenotrophomonas nitritireducens* (1.44) *Stenotrophomonas acidaminiphila* (1.37)	*Achromobacter xylosoxidans*^a^ *Stenotrophomonas rhizophila*	AJ560626.1 MN753976.1	99.87 99.87	740	Species
24	*Testudo graeca*	*Neisseria meningitis* (1.54) *Neisseria elongata* (1.54) *Neisseria subflava* (1.53)	*Uruburuella testudinis*	JX966323.1	100.00	800	Family
25	*Testudo hermanni*	*Neisseria flavescens* (1.51) ^b^ *Neisseria sicca* (1.42) *Neisseria elongata ssp*. *nitroreducens* (1.41)	*Uruburuella testudinis*	JX966328.1	99.38	800	Family

^a^Sequences are suspected to refer to incorrect entries in the database.

^b^Bacterial species appearing twice within the first three MALDI-TOF MS results.

The sequences obtained have been submitted to GenBank and the assigned accession numbers (MT664080-MT664104) are listed in S2 Table: “GenBank accession numbers of sequences obtained in this study in alphabetical order according to bacterial species”.

### Comparison of MALDI-TOF MS and molecular analysis results

Comparison of results gained through 16S rRNA gene sequencing with the results from the first MALDI-TOF MS analysis for each of the isolates assigned to category III showed variable levels of accordance.

#### Gram-positive bacterial isolates

For the 15 gram-positive isolates, the MALDI-TOF MS result corresponded to that of the molecular analysis in one isolate (No. 22) at the species level and in two isolates (Nos. 3, 21) at the genus level. In one case (No. 15) correspondence with molecular analysis result was achieved at the genus level with the result listed thirdly by MALDI-TOF MS. The two methods were in agreement concerning the family level with the results listed secondly and thirdly by MALDI-TOF MS in one case (No. 17). In the remaining cases, the methods did not agree. Three of the isolates in which the two methods did not agree (Nos. 12, 13, 14) were identified as *Devriesea agamarum* by molecular analysis. They were cultured from spiny-tailed lizards (Uromastycinae) and from a Fiji banded iguana (*Brachylophus fasciatus*). The results were all unambiguous in the molecular analysis; however, none of these three isolates was identified correctly within the first three MALDI-TOF MS results. The MALDI-TOF MS scores (1.07, 1.23, 1.17) were also among the lowest achieved in this study. Two further gram-positive isolates (Nos. 15, 16) were classified as belonging to the genus *Exiguobacterium* by molecular analysis. Both derived from chelonians, one from a red-eared slider (*Trachemys scripta elegans*) and the other from an unspecified chelonian species. *Exiguobacterium* sp. was suggested within the first three matches for one of these, but not for the other by MALDI-TOF MS. However, MALDI-TOF MS also suggested *Exiguobacterium aurantiacum* for another isolate, which was later identified as a *Deinococcus* species by 16S rRNA gene sequencing. Two isolates (Nos. 9, 10), cultured from a Mississippi map turtle (*Graptemys pseudogeographica*) and an unspecified chelonian species, were identified as members of the genus *Deinococcus* by molecular analysis. MALDI-TOF MS did not suggest the genus *Deinococcus* within its first three hits for any of these.

#### Gram-negative bacterial isolates

For the ten gram-negative isolates the two identification methods were in agreement to the species level for one isolate (No. 23) and to the genus level for four isolates (Nos. 2, 6, 18, 19). The methods agreed on the family level for two isolates (Nos. 24, 25). MALDI-TOF MS was unable to give any result in the first attempt for two of the gram-negative isolates (Nos. 1, 20). For the final isolate (No. 7) MALDI-TOF MS and the molecular analysis disagreed on the identification.

Gram-negative isolates were identified as *Uruburuella testudinis* in two cases (Nos. 24, 25), obtained from a Hermann’s tortoise (*Testudo hermanni*) and a Greek tortoise (*Testudo graeca*). With the two methods, identification of *Uruburuella testudinis* was only possible based on the 16S rRNA gene sequence. However, the family was identified correctly by MALDI-TOF MS in both cases.

### Incidence of genera of category III isolates among all isolates

The most commonly identified bacterial genera in the samples examined by MALDI-TOF MS were *Pseudomonas* (66 isolates), *Citrobacter* (48 isolates), *Aeromonas* (26 isolates), *Klebsiella* (23 isolates), *Acinetobacter* (21 isolates), *Stenotrophomonas* (18 isolates), and *Staphylococcus* (25 isolates). Several of the bacterial genera identified through 16S rRNA gene sequencing in the category III isolates were also found in a large percentage of other isolates during this study, including *Pseudomonas*, *Staphylococcus*, *Acinetobacter*, and *Stenotrophomonas* (Table 2). Isolates assigned to category III that were found to belong to these genera were all identified correctly at genus level by MALDI-TOF MS independent of their achieved scores. Other genera were only found among the category III isolates, including *Brachybacterium*, *Cloacibacterium*, *Deinococcus*, *Desemzia*, *Devriesea*, *Exiguobacterium*, *Sporosarcina* and *Uruburuella* (Table 2). For some category III isolates assigned to these genera identification was exclusively possible by molecular analysis (Table 1), including species of *Brachybacterium*, *Cloacibacterium*, *Deinococcus*, *Desemzia*, *Devriesea*, and *Exiguobacterium*.

**Table 2:** Numbers of bacteria isolated from skin samples of reptiles from genera containing category III samples in alphabetical order according to genus and numbers of isolates in category III for each.

Genus	Total number of isolates from this genus examined by MALDI-TOF MS	Number of category III isolates (percentage)
*Achromobacter*	5	1 (20.0)
*Acinetobacter*	21	2^a^ (9.5)
*Bacillus*	12	2 (16.7)
*Brachybacterium*	1	1 (100.0)
*Chryseobacterium*	11	2^a^ (18.2)
*Cloacibacterium*	1	1 (100.0)
*Corynebacterium*	2	1 (50.0)
*Deinococcus*	2	2 (100.0)
*Desemzia*	1	1 (100.0)
*Devriesea*	3	3 (100.0)
*Exiguobacterium*	2	2 (100.0)
*Micrococcus*	3	1 (33.3)
*Ochrobactrum*	2	1 (50.0)
*Pseudomonas*	66	1 (1.5)
*Staphylococcus*	25	1 (4.0)
*Serratia*	7	1 (14.3)
*Stenotrophomonas*	18	1 (5.6)
*Sporosarcina*	1	1 (100.0)
*Uruburuella*	2	2 (100.0)
**Totals**	**185**	**27**

^a^No molecular analysis was performed for one isolate later identified as *Chryseobacterium* sp. by MALDI-TOF MS and one isolate identified as *Acinetobacter* sp. with a score of 1.65 due to contamination or a lack of regrowth after deep-freezing.

## Discussion

In veterinary medicine, MALDI-TOF MS is known to be a helpful tool for bacteriological examination [18]. However, for some minor species a significant number of isolates may achieve only low scores or identification by this method may not be possible. This has been previously described for respiratory samples from snakes [23]. Studies evaluating aerobic bacterial isolates from reptile skin that could not be clearly identified by MALDI-TOF MS (low scores of < 1.70) have not been previously reported. MALDI-TOF MS is only one of various tools available for the identification of cultured bacteria. However, in order to evaluate low-scored results, it is crucial to know how often to expect a failed or inaccurate identification and for which bacterial species or even genera identification via MALDI-TOF MS may be problematic.

The majority of the bacterial isolates obtained in this study were identified by MALDI-TOF MS with a score ≥ 2.00 (85.1%) or ≥ 1.70 and < 2.0 (8.4%). This finding is similar to those reported in studies examining bacterial isolates from humans about a decade ago [24,25]. At that time, MALDI-TOF MS was considered an accurate technique, suitable for the identification of bacteria in diagnostic laboratories. Based on the criteria used at that time, it should therefore currently be considered appropriate for identification of bacterial isolates cultured from reptile skin.

In the present study, identification via MALDI-TOF MS was problematic or unfeasible for 6.5% of the isolates found (category III isolates). However, in several of these cases, especially for common gram-negative bacteria, MALDI-TOF MS provided a similar result to that found by 16S rRNA gene sequencing, although identification to the species level was not always possible. Some studies have indicated that MALDI-TOF MS can provide accurate identification of bacteria even in cases in which low scores are achieved [20,24]. Strategies for improving scores in some cases have also been described: duplicate analysis may help decrease the risk of inadequate results, although single spot testing has been determined to be adequate for medical laboratories [25]. Variations in the transfer method used have also been discussed as a factor in the accuracy and robustness of MALDI-TOF MS analysis [21,26] and may influence results in some cases. For low-scored MALDI-TOF MS results retesting—ideally with another transfer method—is therefore obligatory, if no additional techniques are used.

Several isolates were only identified through 16S rRNA gene sequencing in the present study. This was often due to a lack of reference spectra in the database. Many of the bacterial species revealed by molecular analysis in this study are not listed in the MALDI Biotyper Reference Library (MBT Compass Library Revision E, MBT 7854 MSP Library and since December 2019 used Revision F, MBT 8468 MSP Library). This was the case for the species *Bacillus filamentosus*, *Chryseobacterium culicis*, *Deinococcus aquaticus*, *Deinococcus indicus*, *Exiguobacterium acetylicum*, *Micrococcus yunnanensis*, *Ochrobactrum lupini*, *Ochrobactrum pseudogrignonense*, *Pseudomonas reidholzensis*, and *Sporosarcina thermotolerans*. For several other isolates, not even the genus was included in the database. This was the case for the genera *Devriesea*, *Cloacibacterium*, and *Uruburuella*. Therefore, several bacterial species could not be identified based only on MALDI-TOF MS analysis in this study. This highlights the importance of the entries in the selected database, especially when examining bacterial isolates for which insecure results by MALDI-TOF MS cumulate or which are often cultured from uncommon species or locations [21,31]. As is the case for many technical advancements, MALDI-TOF MS was first used in human medicine, and until now, reptiles are not a major focus for available databases, making bacterial diagnostics by MALDI-TOF MS in this group of animals more difficult.

The databases for bacterial identification are expanding rapidly. With the revision (MBT Compass Library Revision F, MBT 8468 MSP Library from 2019) entries for *Desemzia incerta*, and *Brachybacterium conglomeratum* as well as *Brachybacterium paraconglomeratum* have become available. Therefore, isolates of these bacterial species should no longer cause identification problems. However, this update was only used beginning in the last month of this study. Nevertheless, addition of verified spectra into the database by individual laboratories may help to solve most of the identification problems for minor species-derived bacteria faster. The project “MALDI-UP” [32] collects information on generated spectra, classification of the corresponding bacteria as well as the research group responsible for producing the data, and may be helpful in creating new entries in the database.

**Fig 1:**
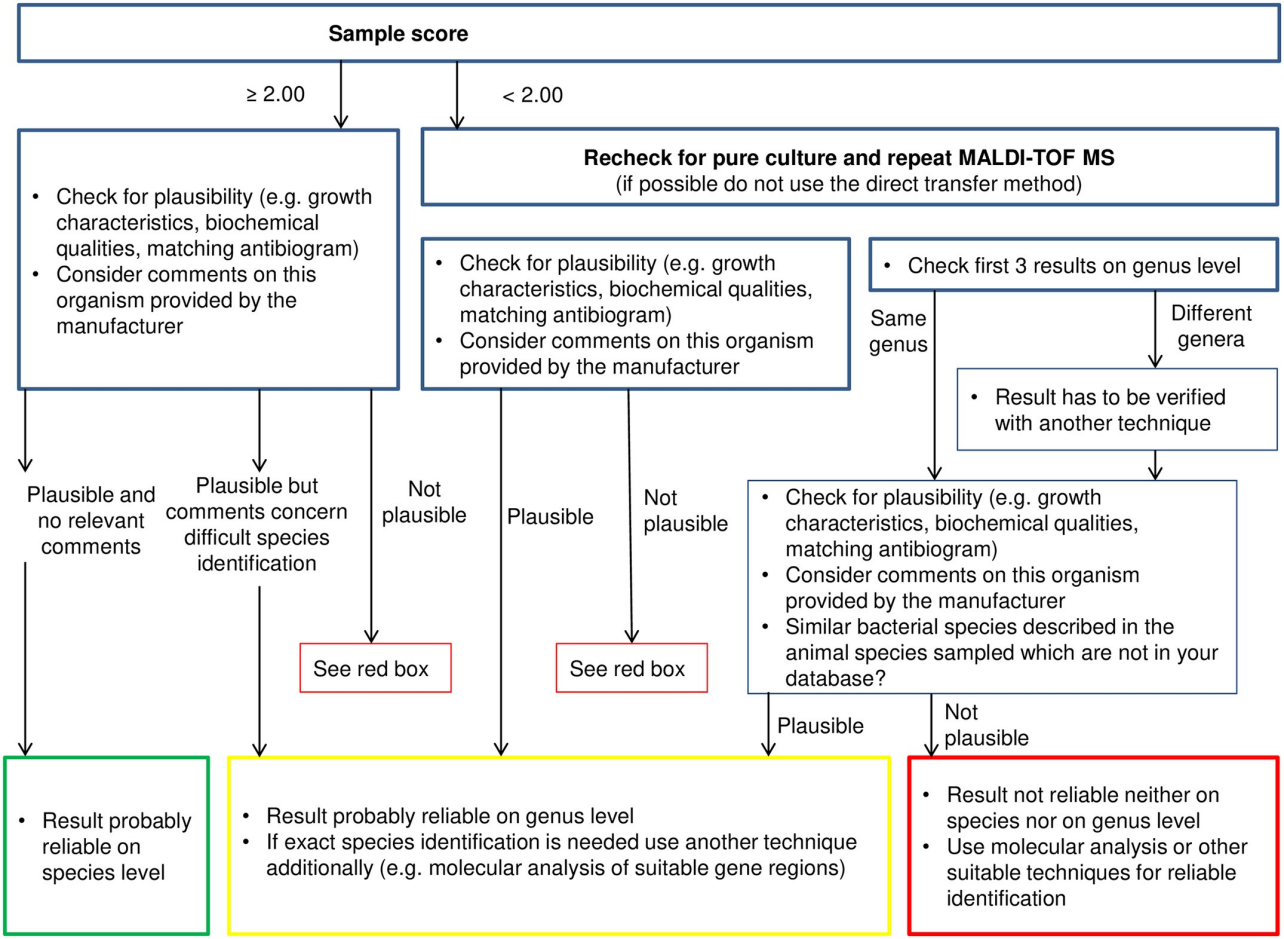
Algorithm for improved interpretation of MALDI-TOF MS generated results for reptile skin-derived aerobic bacterial isolates. Scores refer to scores given by MALDI-TOF MS.

In order to judge the necessity of additional entries, the reliability of generated MALDI-TOF MS results, especially low-scored ones, has to be assessed. An algorithm was therefore developed so as to reliably evaluate aerobic bacteria isolated from reptile skin (Fig 1). It is based on previous studies, which confirmed correct species identification for aerobic bacterial isolates scored ≥ 2.0 and at least correct genus identification for isolates scored ≥ 1.70 in the majority of cases with the mentioned limitations [24,26], as well as on conclusions drawn from the results presented here. This algorithm can be used to help support decision making when working with MALDI-TOF MS. However, even if scored < 1.70 and inconsistent at the genus level with the following results, the first bacterial species given may represent the correct result at species level. Nevertheless, it is highly recommended to reevaluate all results achieving a MALDI-TOF MS score < 1.70 (e.g. with repeated MALDI-TOF MS testing and/or with additional techniques such as 16S rRNA gene sequencing).

It is important to note that 16S rRNA gene sequencing contains some diagnostic pitfalls. Apart from the fact that there is no general guideline for the interpretation of 16S rRNA gene sequence data [8,10], the evaluation of these sequencing results is highly dependent on the database used. A lack of corresponding entries can make identification problematic and the availability of only a limited number of entries for some genera increases the risk of incorrect identifications. While public databases—e.g. GenBank—usually contain huge amounts of entries and therefore are often used by default, they may also include incorrect entries [8,10,14,21]. For instance, this is suspected if one result differs from all others in the BLAST analysis. This was the case in this study for one *Corynebacterium freneyi* sequence between numerous *Corynebacterium xerosis* sequences, for one *Achromobacter xylosoxidans* sequence between numerous *Stenotrophomonas rizophila* sequences and for one *Pantoea* sp. as well as for one *Paenibacillus* sp. sequence of the same submitter between numerous *Ochrobactrum* species sequences.

16S rRNA gene sequencing is known to inadequately discriminate within some genera [8,16], which was also observed in this study, where similar results were obtained for different species of the same genus in some samples. Other gene targets may therefore be more helpful for discriminating between species of the same genus. Nevertheless, the 16S rRNA gene is a useful target for initial identification of bacteria due to its omnipresence in bacterial genomes [10].

Isolates most commonly identified by MALDI-TOF MS in this study belonged to the genera *Pseudomonas*, *Citrobacter*, *Aeromonas*, *Klebsiella*, and *Staphylococcus*. These genera have also been described in previous publications focussing on aerobic bacteria on reptile skin [1,5]. However, in this study, fastidious or slow-growing bacteria may have been overlooked due to the chosen culture conditions (e.g. incubation was carried out only under aerobic conditions and cultures were only incubated for 48 hours if no bacterial growth was observed within the first 18–24 hours) that may not have been suitable for those bacteria.

The low-scored isolates in this study belonged to heterogenic bacterial genera. While some of these genera have only rarely been described, others, such as *Corynebacterium*, *Pseudomonas*, *Serratia*, or *Staphylococcus* are well-known and frequently found in reptile skin lesions or dermal abscesses [1,2,33]. Their clinical relevance is nevertheless not always easy to interpret as it is influenced by many factors. Reptile skin is known to be colonized by various bacterial species acting as commensals or even as a protective part of the microbiome [34]. However, many such bacteria can also infect the skin and cause dermatitis following traumatic lesions or weakened skin integrity, e.g. due to husbandry errors [1,2,35]. Additionally, the clinical relevance of individual bacterial species may not only be influenced by the condition of the skin but also strongly depend on the host species. For instance *Devriesea agamarum* is known to cause dermatitis and has also been linked to sepsis in spiny-tailed lizards (Uromastycinae), whereas bearded dragons (*Pogona* spp.) can be inapparent carriers [36]. It is therefore particularly important that the microbiologist is informed of the host species when evaluating bacteriological results.

Unfortunately, little is known concerning the behaviour of several of the bacterial species or genera identified in this study on reptile skin. However, members of the genera *Uruburuella*, *Exiguobacterium* or *Deinococcus* have been previously isolated from reptiles: *Uruburuella testudinis*, which was isolated from a Greek tortoise and a Hermann’s tortoise in this study, has also been described in tortoises of the genus *Testudo* before [37]. This bacterial species does not seem to be skin-specific and has been isolated from different organs, mainly from the pharynx [37]. In previous studies, two of eleven chelonians infected with *Uruburuella testudinis* were described to be septicaemic, and the closely related species *Uruburuella suis* has been isolated from five pigs with pneumonia and pericarditis [38]. This may indicate an ability of members of the genus *Uruburuella* to be part of systemic infections. However, the available studies are based on only a very small number of cases.

In this study, the two isolates belonging to the genus *Deinococcus* were both derived from chelonians. Members of the genus *Deinococcus* have previously been detected in the skin of healthy snakes and were classified as symbionts [34]. Enzymes from one *Deinococcus* species have also been studied for use in human health care [39]. This genus is therefore unlikely to be harmful for the skin.

Two chelonians in this study were found to host members of the genus *Exiguobacterium*. *Exiguobacterium* spp. have been isolated from various sources, including from the cloaca of Chinese alligators [40] and numerous environmental sources [41]. To our knowledge, there are no case reports describing any clinical relevance of this genus in animals. In human medicine, these bacteria have only rarely been described in conjunction with clinical afflictions [42,43]. Their ability to affect human skin has been discussed controversially [44].

The fact that clinical relevance of bacterial species found on reptile skin is often unclear, stresses the importance of a systematic anamnesis with special regard to underlying husbandry issues [1,2,45] and a detailed clinical examination of the patient. Also, further research, based on correct identification of the bacterial species, is needed. For this, MALDI-TOF MS is a suitable tool if the databases continue to be extended regularly.

## Conclusion

MALDI-TOF MS is a suitable tool for the identification of most bacterial isolates cultured from reptile skin. However, there remain holes in the available databases for the exact identification of some bacteria found in these animals. Common genera like *Pseudomonas*, *Acinetobacter* or *Staphylococcus* seem to have a lower risk of inaccurate identification at the genus level than less common bacteria, which may not yet be included in the databases. It is necessary to carefully examine the plausibility of low-scored MALDI-TOF MS results (e.g. using growth characteristics, biochemical qualities, matching antibiograms). It is also helpful to be familiar with the occurrence, the morphology, and biochemical characteristics of some reptile-specific bacterial species (e.g. *Devriesea agamarum* or *Uruburuella testudinis*) and with the limits of the database used. Examination quality can be improved by the addition of reliable entries in the MALDI-TOF MS database. Supplementary methods like 16S rRNA gene sequencing should be used to aid in identification of isolates whenever plausibility testing is not successful and are recommended for isolates that achieved a MALDI-TOF MS score < 1.70.

## Supporting information

S1 Table Supplemental information on host species in alphabetical order. (DOCX)

S2 Table GenBank accession numbers of sequences obtained in this study in alphabetical order according to bacterial species. (DOCX)
