# Supplementary material for: Challenges in microbiological identification of aerobic bacteria isolated from the skin of reptiles
Source: PLoS One. 2020 Oct 19;15(10):e0240085. doi: 10.1371/journal.pone.0240085 (PMC7571677; doi:10.1371/journal.pone.0240085)
Supplement: S1 Table — (DOCX) [file pone.0240085.s001.docx]

Table S1: Supplemental information on host species in alphabetical order

| **Group** | **Species** | **Total number** |
| --- | --- | --- |
| **Chelonians** |  | **136** |
|  | *Aldabrachelys gigantea* | 1 |
|  | *Caretta caretta* | 4 |
|  | *Chelonia mydas* | 2 |
|  | Cheloniidae (species unknown) | 1 |
|  | *Chelonoidis carbonarius* | 2 |
|  | *Chelydra serpentina* | 1 |
|  | *Elseya branderhorsti* | 1 |
|  | Emydidae (species unknown) | 1 |
|  | *Emydura subglobosa* | 3 |
|  | *Emys orbicularis* | 1 |
|  | *Eretmochelys imbricata* | 1 |
|  | *Graptemys pseudogeographica* | 9 |
|  | *Graptemys* sp. (species unknown) | 2 |
|  | *Mauremys leprosa* | 1 |
|  | *Mauremys reevesii* | 3 |
|  | *Pelodiscus sinensis* | 1 |
|  | *Pelomedusa subrufa* | 1 |
|  | *Pseudemys nelsoni* | 1 |
|  | *Pseudemys* sp (species unknown) | 1 |
|  | *Rhinoclemmys pulcherrima manni* | 1 |
|  | *Sternotherus odoratus* | 8 |
|  | *Testudo graeca* | 6 |
|  | *Testudo hermanni* | 19 |
|  | *Testudo marginata* | 3 |
|  | *Trachemys scripta* (subspecies unknown) | 7 |
|  | *Trachemys scripta elegans* | 8 |
|  | *Trachemys scripta scripta* | 6 |
|  | *Trachemys scripta troostii* | 1 |
|  | *Trachemys* sp. (species unknown) | 4 |
|  | Unknown | 36 |
| **Squamates (excluding snakes)** |  | **61** |
|  | *Brachylophus fasciatus* | 2 |
|  | *Chamaeleo calyptratus* | 5 |
|  | *Chamaeleo melleri* | 1 |
|  | Chamaeleonidae (species unknown) | 1 |
|  | *Ctenosaura* sp. (species unknown) | 1 |
|  | *Corytophanes cristatus* | 1 |
|  | *Egernia stokesii* | 1 |
|  | *Eublepharis macularius* | 5 |
|  | *Furcifer pardalis* | 3 |
|  | *Gekko gecko* | 1 |
|  | *Iguana iguana* | 7 |
|  | *Iguana* sp. (species unknown) | 2 |
|  | Iguanidae (species unknown) | 3 |
|  | *Lacerta bilineata* | 1 |
|  | *Paleosuchus palpebrosus* | 1 |
|  | *Physignathus coccincinus* | 1 |
|  | *Pogona henrylawsoni* | 2 |
|  | *Pogona* sp. (species unknown) | 5 |
|  | *Pogona vitticeps* | 1 |
|  | *Tiliqua rugosa* | 1 |
|  | Uromastycinae (species unknown) | 4 |
|  | *Varanus cumingi* | 1 |
|  | *Varanus gilleni* | 1 |
|  | *Varanus glauerti* | 2 |
|  | *Varanus indicus* | 1 |
|  | Unknown | 7 |
| **Snakes** |  | **38** |
|  | *Boa constrictor* | 4 |
|  | Boidae (species unknown) | 2 |
|  | *Crotalus viridis* | 1 |
|  | *Elaphe schrenckii* | 1 |
|  | *Epicrates* sp. (species unknown) | 1 |
|  | *Eunectes murinus* | 2 |
|  | *Gonyosoma prasinum* | 1 |
|  | *Lampropeltis* sp. (species unknown) | 1 |
|  | *Liasis fuscus* | 1 |
|  | *Morelia spilota* | 1 |
|  | *Natrix tessellata* | 1 |
|  | *Pantherophis guttatus* | 6 |
|  | *Python molurus* | 1 |
|  | *Python regius* | 7 |
|  | Pythonidae (species unknown) | 4 |
|  | *Thamnophis* sp. (species unknown) | 1 |
|  | *Vipera aspis* | 1 |
|  | Unknown | 2 |
