# Supplementary material for: Challenges in microbiological identification of aerobic bacteria isolated from the skin of reptiles
Source: PLoS One. 2020 Oct 19;15(10):e0240085. doi: 10.1371/journal.pone.0240085 (PMC7571677; doi:10.1371/journal.pone.0240085)
Supplement: S2 Table — (DOCX) [file pone.0240085.s002.docx]

Table S2: GenBank accession numbers of sequences obtained in this study in alphabetical order according to bacterial species

| **Bacterial species** | **Accession number** |
| --- | --- |
| *Achromobacter xylosoxidans* | MT664080 |
| *Acinetobacter haemolyticus* | MT664081 |
| *Bacillus* sp. | MT664082 |
| *Bacillus* sp. | MT664083 |
| *Brachybacterium* sp. | MT664084 |
| *Chryseobacterium culicis* | MT664085 |
| *Cloacibacterium normanense* | MT664086 |
| *Corynebacterium xerosis* | MT664087 |
| *Deinococcus aquaticus* | MT664088 |
| *Deinococcus indicus* | MT664089 |
| *Desemzia* sp. | MT664090 |
| *Devriesea agamarum* | MT664091 |
| *Devriesea agamarum* | MT664092 |
| *Devriesea agamarum* | MT664093 |
| *Exiguobacterium acetylicum* | MT664094 |
| *Exiguobacterium* sp. | MT664095 |
| *Micrococcus* sp. | MT664096 |
| *Ochrobactrum* sp. | MT664097 |
| *Pseudomonas* sp. | MT664098 |
| *Serratia marcescens* | MT664099 |
| *Sporosarcina thermotolerans* | MT664100 |
| *Staphylococcus kloosii* | MT664101 |
| *Stenotrophomonas rhizophila* | MT664102 |
| *Uruburuella testudinis* | MT664103 |
| *Uruburuella testudinis* | MT664104 |
